# A Case Report of Myxedema Coma in the Setting of Normal Thyroid Stimulating Hormone

**DOI:** 10.7759/cureus.62695

**Published:** 2024-06-19

**Authors:** Klynt Bally, Rhonda-Kaye Trusty, Kamrun Naher

**Affiliations:** 1 Internal Medicine, State University of New York Downstate Medical Center, New York City, USA; 2 Internal Medicine and Endocrinology, State University of New York Downstate Medical Center, New York City, USA; 3 Endocrinology, State University of New York Downstate Medical Center, New York City, USA

**Keywords:** thyroid disease, normal tsh, myxedema coma, levothyroxine, hypothyroidism

## Abstract

Myxedema coma (MC) is a potentially fatal complication of hypothyroidism, with a high mortality rate. It is a clinically diagnosed condition, where the symptoms are related to decreased metabolic effects due to low active thyroid hormones. This case report highlights a severe case of MC, despite the thyroid stimulating hormone (TSH) being normal and the free thyroxine (FT4) being very mildly decreased.

## Introduction

Myxedema coma (MC) has an incidence of approximately 0.22 per 1000000 per year based on a study published in 2023 [1]. It is a clinically diagnosed condition, where the symptoms are related to decreased metabolic effects due to low active thyroid hormones. This case report highlights a severe case of MC, despite thyroid stimulating hormone (TSH) being normal and a very mildly decreased free T4 (FT4), reiterating that the severity of the disease does not correlate with the degree of derangement of thyroid function tests (TFTs).

## Case presentation

A 95-year-old female with hypothyroidism (compliant on levothyroxine (LT4)), Alzheimer’s disease, type 2 diabetes, and hypertension, presented for one day of altered mental status. Her baseline was conversational, but bed-bound. She was noted to be non-verbal and lethargic that morning. Of note, the patient had pneumonia two weeks prior and was treated with a course of doxycycline. It was also revealed that the patient was taking her LT4 along with her other chronic medications (donepezil, rosuvastatin, metformin and quetiapine). On presentation, vital signs were notable for bradycardia (between 40-60 beats per minute), hypothermia (88.7°F) and hypotension. The initial exam was positive for disorientation, cold skin, hyporeflexia and bradycardia. Labs showed hyponatremia 124 mEq/L, respiratory acidosis, normal TSH (3.190 µIU/mL) and very mildly decreased FT4 (0.92 ng/dL, range 0.93-1.70 ng/dL). Infection was ruled out. CT head revealed no abnormalities. The intensive care unit was consulted, a norepinephrine drip was started, and the patient was intubated due to the inability to protect the airway in the setting of her altered mental status. The endocrinology team was consulted for high suspicion of MC (with an MC scale score of 115). Adrenal insufficiency (AI) was never sufficiently ruled out due to being treated with steroids prophylactically. The patient was treated with intravenous (IV) LT4 and hydrocortisone, and mental status returned to baseline, with maintained normal TSH and normalized FT4 (refer to Table 1). In the clinic, the patient was maintained with normal TSH and FT4 and due to the ambiguity of AI, steroids were continued.

**Table 1:** Thyroid Function Tests During Hospitalization

Test	Reference Range	Day 1	Day 2	Day 3	Day 5	Day 6	Clinic Post-discharge
Free Thyroxine	0.93-1.70 ng/dL	0.92	1.56	1.28	1.21	1.46	1.27
Thyroid Stimulating Hormone	0.270-4.200 µIU/mL	3.190	1.710	0.649	0.499	0.409	0.928
Thyroid Peroxidase	<=34.9 IU/mL	933					

## Discussion

Hypothyroid patients already have compensatory/homeostatic mechanisms to overcome the lack of metabolic effects due to decreased thyroid hormone levels. However, in the setting of various triggers/stressors, these compensatory mechanisms are overwhelmed, requiring a higher need for thyroid hormones, eventually leading to hemodynamic collapse and clinical symptoms of MC. Triggers for MC include myocardial infarction, sepsis, trauma, anaesthesia, or stroke, with uncommon causes such as haloperidol [2], and in this case, the trigger is ineffective absorption of her LT4 due to taking it along with her other chronic medications.

Timing for LT4 absorption is specific, needing to be taken on an empty stomach due to increased and effective absorption while fasting [3], whereas the timing still being arguable with one study showing improved thyroid hormone levels with evening dosing of LT4 [4]. In a review published in 2017, studies that investigated the absorption of LT4 in the setting of different medications, disorders, and foods were highlighted and found that the majority of interactions were significant [5]. This is important as patients on LT4 should be counselled and closely monitored to ensure appropriate absorption by monitoring their TFTs, an intervention that could’ve prevented our patient’s MC. Without an elevated FT4, it is very unlikely for MC to be present and other diagnoses should be considered. TSH is almost always elevated in all cases of MC based on literature review, making this case even more compelling.

There have been cases, such as this one, that showcase severe symptoms being produced even with just the slightest abnormalities in TFTs. Two case reports previously published support this, with both presenting with subclinical hypothyroidism with severe circulatory collapse and coma. One presented with FT4 10.7 pmol/L (reference range 10.3-24.5), thyrotropin 6.09 mU/L (reference range 0.4-4.0) with unknown trigger [6], and another with labs notable for TSH (59.9) and FT4 (0.82, reference range being 0.78-2.19), in the setting of pneumonia [7]. In both these cases, the patients were also upgraded to the intensive care unit for further care.

What makes our case even more unique is that our patient was not in subclinical hypothyroidism, but her TSH and FT4 were relatively normal and her clinical status improved dramatically with hydrocortisone and IV LT4. This case presentation and discussion signify the importance of understanding that MC should remain a clinical diagnosis and that TFTs may not be needed to confirm the disease.

## Conclusions

Many providers rely on TFTs to help make the diagnosis of MC. However, this case teaches that MC should not be excluded in the setting of normal/minimally skewed TFTs and should continue to be a clinical diagnosis. Additionally, patients should be counselled extensively on the appropriate administration of LT4 to prevent MC, including waiting three to four hours before taking specific medications such as iron, calcium, and multivitamins.
